# Towards Optimizing Width Modulation for Maximum Thermoelectric Efficiency

**DOI:** 10.3390/mi14122176

**Published:** 2023-11-29

**Authors:** Antonios-Dimitrios Stefanou, Xanthippi Zianni

**Affiliations:** Department of Aerospace Science and Technology, School of Science, National and Kapodistrian University of Athens, 34400 Psachna, Greece; antstefanou@aerospace.uoa.gr

**Keywords:** width-modulated nanowaveguides, metamaterials, electron transport, phonon transport, thermal conduction, thermoelectric efficiency

## Abstract

Maximizing thermoelectric efficiency is typically addressed as identical to minimizing parasitic thermal conduction. Such an approach relies on the assumption that the adopted strategy mainly affects phonons, leaving electrons intact, and is not justified in many cases of non-uniform nanostructures such as width-modulated nanowaveguides, where both electrons and phonons are significantly affected by width modulation. Here, we address the question of maximizing the thermoelectric efficiency of this class of metamaterials by exploring the effect of the modulation extent on both electron and phonon transport. We investigated the effect of increasing modulation degree on the thermoelectric efficiency, considering the cases of (a) a two-QD modulation and (b) multiple-QD modulations in periodic and aperiodic sequences. We show that the thermoelectric efficiency depends on the coupling between the modulation units and the interplay between periodicity and aperiodicity in the modulation profile. We reveal that the maximization of the thermoelectric power factor is for periodic width-modulation, whereas the maximization of the thermoelectric efficiency is for aperiodic width-modulation profiles that form quasi-localized states for electrons. Our work provides new insight that can be used to optimize width modulation for maximum thermoelectric efficiency.

## 1. Introduction

The enhancement of thermoelectric (TE) efficiency has been the subject of intensive research for several decades. It is still a timely and crucial task because if accomplished, it would greatly benefit the current needs of our society for energy production and efficient nanoelectronics applications. The low TE efficiency of traditional TE materials kept their applications limited. The scientific community reorientated research towards new-material and structural engineering approaches. Much effort has been devoted to enhancing the TE efficiency by modifying electrons and phonons by engineering the composition of bulk materials or taking advantage of quantum confinement in low-dimensional materials and heterostructures. The inadequate control of the transport properties of nanostructures still restricts the development of efficient TE applications. An alternative strategy was proposed to use metamaterials [1]. Metamaterials are artificial structures with properties dominated by their geometry and have revolutionized many fields of modern technology such as photonics, acoustics, sensing, etc. [2,3,4]. They could also work for thermoelectrics because thermal and electrical transport properties can be geometrically controlled. Thermoelectric metamaterials (THEMMs) are nanowaveguides (NWs) with enhanced thermoelectric properties controlled by geometry modulation. It has been theoretically demonstrated that the TE properties of width-modulated NWs are strongly dependent on their modulation profile and are candidates for high TE efficiency [5,6]. Their operation relies on the same physics principles as low-dimensional structures [7,8,9,10,11,12,13,14]. Good TE properties stem from two sources: (i) the quantum confinement of carriers (electrons and phonons) restricted in small dimensions and occupying energy states modified from bulk, and (ii) enhanced scattering at geometrical discontinuities, boundaries, and interfaces that limit thermal conduction.

A measure of the TE efficiency is the dimensionless figure of merit ZT defined as ZT = σS^2^T/(k_ph_ + k_e_), where σ is the electrical conductivity, S is the Seebeck coefficient, and k_ph_ and k_e_ are the phonon and electron thermal conductivities, respectively. A good TE material should have good electrical conductivity and a high Seebeck coefficient to provide high TE power (high σS^2^). Moreover, it should be a bad heat conductor (with low k_ph_ and k_e_) to minimize parasitic heat flow from the hot to the cold contact. Achieving simultaneous good electrical transport properties, high TE power, and poor thermal properties is challenging because these transport properties are interdependent. Typically, mechanisms that decrease thermal conduction also decrease electron conduction and deteriorate the TE power. This problem was encountered in low-dimensional material structures and is also expected in width-modulated nanowaveguides (MNWs). However, MNWs have an additional advantage: the ability to geometrical control the transport properties. It has been shown that the shape of the modulation profile drastically affects electron and phonon transport [5] and that controlled disorder in the modulation profile can result in high TE power, low thermal conduction, and overall enhancement of the TE efficiency [6]. The TE efficiency could be maximized by optimizing the modulation profile of MNWs.

Geometry modulation can be realized in multiple ways and with variable degrees of complexity. Designing geometry modulation for optimal thermal and electric transport is a very challenging task. The optimization of the modulation profile of MNWs for minimum phonon thermal conduction has been previously addressed. Phonon conduction was found to decrease monotonically with increasing disorder in the width-modulation profile of aperiodic NWs [5,6]. Optimal width modulation occurs for maximum disorder in the modulation profile, i.e., the maximum number of non-identical modulation units (QDs). The problem of optimization of the modulation profile of MNWs for maximum TE efficiency has not been adequately addressed. It cannot be trivially answered by optimization for minimum phonon conduction because the simultaneous effect on electron transport properties cannot be neglected. Our recent study on the effect of width mismatch on the TE efficiency of an NW modulated by a single modulation unit indicated distinct behaviors for electrons and phonons [15]. We pointed out that the electron thermoelectric power factor shows a non-monotonic dependence, whereas phonon thermal conductance decreases monotonically with the increasing width mismatch of the modulation unit. The optimization of the modulation profile for maximum TE efficiency would require an understanding of electron and phonon transport in the presence of multiple modulation units. The objective of the present work is to address this question. For this, we investigated the effect of increasing the modulation degree considering the cases of (a) two-QD modulation and (b) multiple-QD modulation in periodic and aperiodic sequences and provide new evidence on additional underlying mechanisms. Our results show that the thermoelectric efficiency depends on the coupling between the modulation units and the interplay between periodicity and aperiodicity in the modulation profile. It is revealed that the maximization of the thermoelectric power factor is for periodic width modulation, whereas the maximization of the thermoelectric efficiency is for aperiodic width-modulation profiles that form quasi-localized states for electrons. In Section 2, we define the structures under investigation and detail our methodology. In Section 3, we present and discuss our findings. In Section 4, we draw our conclusion.

## 2. Theoretical Model and Methodology

**Figure 1:**
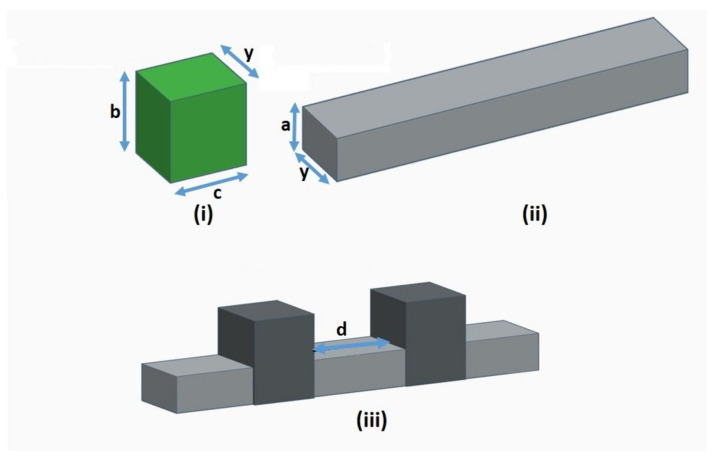
Schematic representation of the structures of interest: (**i**) modulation unit (QD) with width b, length c, and depth y; (**ii**) uniform NW of width a and depth y; (**iii**) a two-QD modulation configuration.

**Figure 2:**
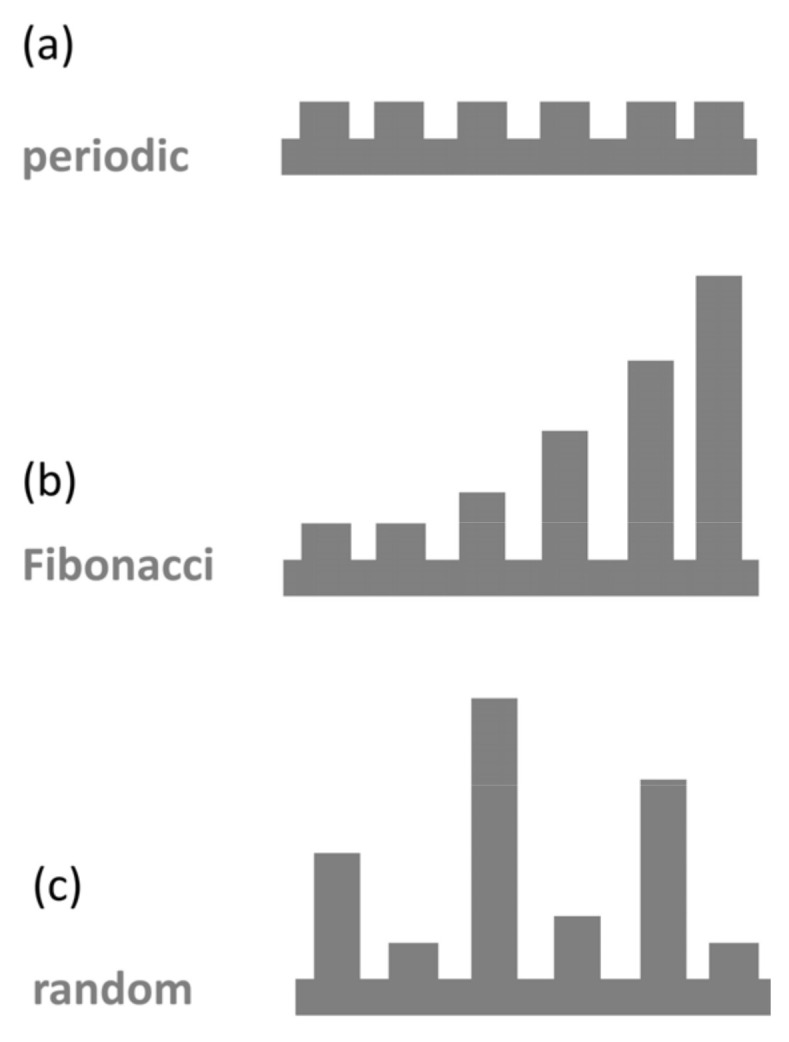
Schematic representation of the multiple-QD modulation configurations: (**a**) the periodic SL, (**b**) the Fibonacci sequence, (**c**) the random sequence.

We consider MNWs modulated by a sequence of modulation units (Figure 1 and Figure 2). Each structure can be thought of as emerging from coupling between an infinite NW and wider finite segments (QDs). Rectangular cross-sections of MNWs have been chosen as suitable reference structures [1]. We present representative results for GaAs NWs of width a = 10 nm and depth y = 12 nm modulated by QDs of width b = 20 nm and length c = 20 nm. We consider the cases of (a) two-QD modulation and (b) multiple-QD modulation. In case (a), the QD separation takes variable values in the range of 5–120 nm (Figure 1). In case (b), we consider sequences of six QDs with constant y = 12 nm, c = 20 nm, and d = 20 nm that are either periodic with fixed b (b = 20 nm) or aperiodic with variable b. The values of b in the aperiodic sequences are 10, 10, 20, 30, 40, 50, and 80 nm. We consider two cases of sequences: an ordered Fibonacci sequence and a random sequence (Figure 2).

We work in the ballistic transport regime, which is suitable for studying the effects of geometry modulation on electron and phonon states as well as on transport unscreened by effects due to additional scattering mechanisms [16,17]. We used Landauer formalism to calculate the transport properties of electrons and phonons in terms of the transmission coefficient [1,5,18,19,20,21]. The transmission coefficient was calculated using scattering matrix theory [22]. We keep our study at low temperatures to avoid the thermal broadening of quantum confinement effects on transport [23,24]. We used the same theoretical model as in Ref. [15]; however, we include a description here for completeness.

The thermoelectric efficiency is estimated by the dimensionless figure of merit ZT:

(1) $$\mathrm{ZT}=\frac{S^{2}\mathrm{GT}}{\kappa }$$

where T is the absolute temperature, G is the electron conductance, and S is the Seebeck coefficient. The numerator GS^2^T is the thermoelectric power factor (TPF). The denominator k is the total thermal conductance expressed as the sum of the electron and the phonon thermal conductances, k_e_ and k_ph_, respectively:

(2) $$\kappa =\kappa_{e}+\kappa_{\mathrm{ph}}$$

An optimal figure of merit ZT_0_ can be defined by neglecting the phonon thermal conductance k_ph_ in Equations (1) and (2).

The electron conductance G, the thermal coefficient K, and the Seebeck coefficient S are given by the following expressions:

(3) $$G=-\frac{2e^{2}}{h}\int \mathrm{dETe}\left(E\right)\frac{\partial f}{\partial E}$$

(4) $$S=-\frac{1}{\mathrm{eT}}\frac{\int \mathrm{dE}\left(E-E_{F}\right)\mathrm{Te}\left(E\right)\frac{\partial f}{\partial E}}{\int \mathrm{dETe}\left(E\right)\frac{\partial f}{\partial E}}$$

(5) $$K=\frac{2e^{2}}{h}\frac{1}{e^{2}T}\int \mathrm{dE}{\left(E-E_{F}\right)}^{2}\mathrm{Te}\left(E\right)\frac{\partial f}{\partial E}$$

In the above equations T_e_(E) stands for the electron transmission coefficient, and E_F_ for the electron Fermi energy.

The electron thermal conductance k_e_ is calculated in terms of the above coefficients using the expression:

(6) $$\kappa_{e}=-K-S^{2}\mathrm{GT}$$

In figures, the electron conductance is expressed in the unit of the conductance quantum $\frac{2e^{2}}{h}$ and the electron thermal conductance in the unit of the thermal conductance quantum $\kappa_{0}=\frac{\pi^{2}k_{b}^{2}}{3h}$.

The phonon thermal conductance k_ph_ in terms of the phonon transmission coefficient is calculated by the following expression:

(7) $$\kappa_{\mathrm{ph}}=\frac{h^{2}}{k_{B}T}\underset{m}{\sum }\frac{1}{2\pi }\int_{\omega_{m}}^{\omega }T_{m}\left(\omega \right)\frac{\omega^{2}e^{\hbar \omega /k_{B}T}}{{\left(e^{\hbar \omega /k_{B}T}-1\right)}^{2}}d\omega$$

The quantity ω_m_ is the cut-off frequency of an m mode, and the transmission coefficient for phonon mode m versus the frequency ω is expressed as T_m_ (ω). The total transmission coefficient of phonons can be calculated as the total sum of the phonon modes m for frequency ω, as follows:

(8) $$T_{\mathrm{ph}}\left(\omega \right)=\underset{m}{\sum }T_{m}\left(\omega \right)$$

The phonon frequency ω is shown as follows in figures in units of Δ:

(9) $$\;\Delta =\omega_{m+1}-\omega_{m}=\pi u/a$$

where Δ is the cut-off frequency’s splitting between an m + 1 and an m mode, while a is the width of the nanowire, and u is the sound velocity. In the figures, the phonon thermal conductance is expressed in units of κ_0_.

## 3. Results and Discussion

Electron and phonon transport properties depend strongly on the modulation profile of MNWs [1,5]. Systematic calculations have shown that phonon transmission and phonon thermal conductance decrease systematically with the increasing modulation degree quantified by the number and sizes of the modulation units [5,6]. This is due to more extended destructive interference between scattered phonon waves at more discontinuities when the number of modulation units increases. Here, we focus on the dependence of the electron transport properties and the TE efficiency on the modulation degree. In a recent study, we explored the effect of a single-QD modulation and showed that the TE efficiency depends non-monotonically on the width mismatch of a single modulation unit [15]. We have extended our study on the effects of (a) a two-QD modulation and (b) a multiple-QD modulation. We present and discuss our results in the two sub-sections that follow. Similarly, as in the case of the single modulation unit, we plot our calculations on the transport properties of electrons and phonons and on the TE efficiency metrics for the actual relevant parameters. It will be made apparent that the results indicate distinct underlying mechanisms in the two studies. In the case of the single modulation unit, the effect of the width mismatch is interpreted by quantum confinement. In the present case of multiple-unit modulation, the results will be interpreted by the coupling between the modulation units and the interplay between order and disorder in the array of the modulation units.

### 3.1. The Effect of a Two-QD Modulation

**Figure 3:**
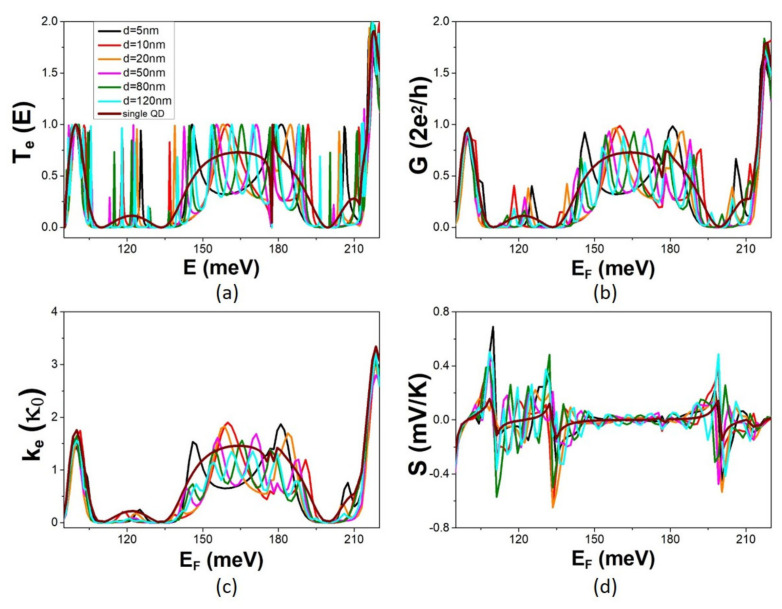
The electron transport properties in the case of two-QD modulation for variable QD separation d: (**a**) the energy dependence of the electron transmission coefficient T_e_, and (**b**–**d**) the dependence of the conductance G, the electron thermal conductance k_e_, and the Seebeck coefficient S, respectively, on the Fermi energy E_F_ calculated at T = 5 K. The dimensions are defined in text. Calculations for the single-QD modulation are shown for reference.

We consider a NW modulated by two QDs in separation d (Figure 1). We fixed the dimensions of each QD, varying the separation d between the two QDs and calculated the electron transmission coefficient T_e_(E) and transport properties G, S, and k_e_. Representative calculations for d in the range of 10–120 nm are shown in Figure 3 together with the corresponding ones for a single-QD modulation that serves as a reference in our discussion. The transport properties are determined by T_e_(E) according Landauer formalism as detailed in Section 2. The transmission coefficient of a MNW fluctuates with energy showing characteristic peaks, deeps, and zeros resulting from interference between waves propagating across the NW with waves scattered at the width discontinuities. Destructive interference decreases conductance below that of the perfect NW. The deeps of T_e_(E) of the NW modulated by a single QD correspond to the QD energy states [1,5]. They result from completely destructive interference between the NW waves and the waves scattered at the width discontinuities because of mismatch between their energies and the QD energies. The zeros of T_e_(E) are for completely destructive interference. Two subsequent transmission zeros define a conduction miniband. A comparison of the transmission coefficients of NWs modulated by a single QD and by two QDs shows that the single-QD modulation minibands remain present when a second QD is added in the modulation profile irrespective of the separation between the QDs. The transmission coefficient of a two-QD MNW shows additional fluctuations within each of these minibands that are attributed to the coupling between the two QDs. The fluctuations change upon changing the QD separation d because the coupling between the QDs changes. For smaller separation d, the coupling is stronger, the QD states repeal more significantly, and their energy separation increases. This is why fluctuations are sharper for small d and shallower for increasing d. For increasing d, the coupling between the QDs is weaker, and the QD states repeal less and are closer in energy, resulting in a denser spectrum of peaks and shallower fluctuations. The electron conductance G and the thermal conductance k_e_ follow the energy dependence of the transmission coefficient showing the same fluctuations with the Fermi energy E_F_. Conduction fluctuations are thermally broadened with increasing temperature. Thus, we refer to calculations at low temperatures (T < 10 K) where quantum confinement effects dominate transport and are not screened by thermal broadening. The Seebeck coefficient shows peaks at the conduction thresholds and at the conduction fluctuations. This is expected by the fact that that the Seebeck coefficient depends on the rate of change of the conductance [16]. The highest peaks are observed at the thresholds of the single-QD modulation minibands because they are sharper than at conduction fluctuations within the minibands. Notably, the peaks at the minibands thresholds are much higher for the two-QD modulation than for the single-QD modulation.

**Figure 4:**
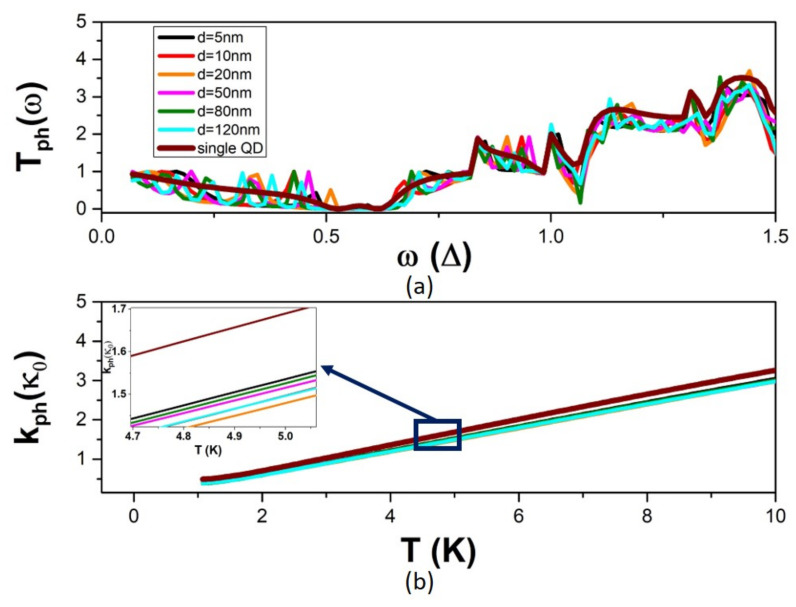
The phonon transport properties in the case of two-QD modulation for variable QD separation d: (**a**) the frequency dependence of the phonon electron transmission coefficient T_ph_, and (**b**) the temperature dependence of the phonon thermal conductance k_ph_. Calculations for the single-QD modulation case are included for reference. The inset zooms in curves for different values of d.

The calculated phonon transmission coefficient T_ph_ and the phonon thermal conductance k_ph_ for QD modulation and the single-QD modulation are shown in Figure 4. As in the case of electrons, the destructive quantum interference of phonon waves at width discontinuities decreases phonon transmission below that of the uniform NW. The decrease is dominated by the single-QD modulation. The T_ph_ of the two-QD modulation shows additional secondary fluctuations around the T_ph_ of the single-QD modulation. These fluctuations have a weak average effect on k_ph_, which consequently shows a small decrease upon the addition of a second QD in the modulation profile. The dependence of k_ph_ on the QD separation d is also small. The inset of Figure 4b zooms in k_ph_ for different values of d. It shows the non-monotonic dependence of k_ph_ on d, which reflects the non-regular quantum interference between multiple phonon modes.

**Figure 5:**
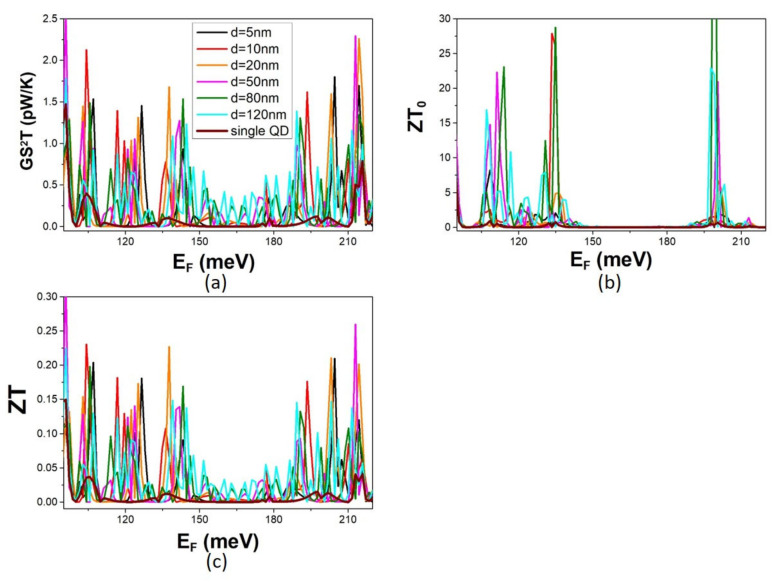
The thermoelectric efficiency in the case of two-QD modulation for variable QD separation d. The Fermi energy, E_F_; the dependence of (**a**) the thermoelectric power factor (TPF), (**b**) the optimal figure of merit ZT_0_, and (**c**) the figure of merit ZT calculated at T = 5 K for the structures defined in text. Calculations for the single-QD modulation are shown for reference.

The TE efficiency is shown in Figure 5. The TPF is significantly enhanced in the two-QD modulation compared to the single-QD modulation. It shows high peaks at conduction minibands and additional peaks within minibands. The peaks of the TPF at the thresholds of the minibands are significantly enhanced compared to the single-QD modulation due to the corresponding enhancement of the Seebeck coefficient. The enhancement is bigger at narrower minibands as expected by the corresponding bigger enhancement of the Seebeck coefficient. The highest TPF peaks are observed for a smaller QD separation d when the QD coupling is stronger. The TPF peaks decrease in height and increase in density with increasing d. They become shallower fluctuations for weaker coupling between the QDs. The optimal figure of merit ZT_0_ shows an enhancement similar to that of the TPF. The peaks of ZT_0_ are very high at narrow minibands because the electron thermal conductance is asymptotically small. The peaks of the figure of merit decrease when phonon thermal conductance is also taken into account. The peaks of the ZT are lower than those of the ZT_0_ because phonon thermal conduction dominates thermal conduction. The ZT follows the same Fermi energy E_F_ dependence as the TPF. It can be concluded that the TE efficiency increases with the addition of a second QD in the modulation profile. The enhancement is bigger for stronger coupling between the two QDs. The increase in the TE efficiency is dominated by the effect of QD coupling on electron transport.

### 3.2. The Effect of Multiple-QD Modulation

Next, we consider arrays of multiple QDs in the modulation profile. We fix the dimensions of the QDs and their separation d. We chose d = 20 nm because coupling between the QDs is significant as shown in the calculations of the previous sub-section. We discuss calculations on a sequence of six identical QDs as representative of the infinite SL based on previous evidence that the perfect SL is well approximated for a minimum of five QDs [1,5]. We explore the effect of disorder in the modulation profile by comparing the perfect SL with aperiodic arrays of the same number of QDs in Fibonacci and in random arrangements.

**Figure 6:**
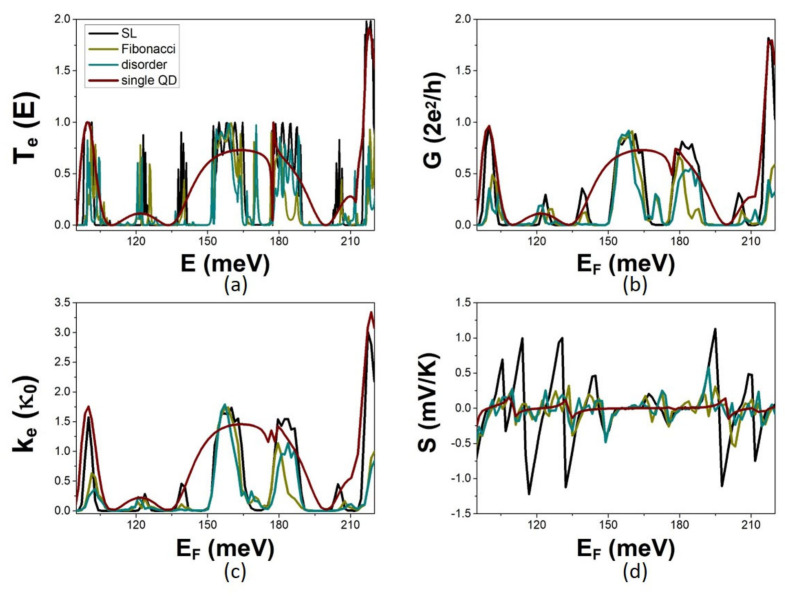
The electron transport properties in the case of multiple-QD modulation for the SL, the Fibonacci and the random sequences as described in text. (**a**) The energy dependence of the electron transmission coefficient T_e_, and (**b**–**d**) the energy Fermi, E_F_, dependence of the conductance G, the electron thermal conductance k_e_, and the Seebeck coefficient S, respectively, calculated at T = 5 K for dimensions defined in text. Calculations for the single-QD modulation are shown for reference.

Calculated electron transmission coefficient T_e_(E) and transport properties G, S, and k_e_, are shown in Figure 6 together with the corresponding ones for the reference single-QD modulation. The SL transmission coefficient shows a much richer energy spectrum with more minibands than the single-QD modulation transmission coefficient. The formation of the SL minibands is the outcome of constructive interference between the states of the identical QDs in the periodic array that have the same symmetry. The SL minibands are narrower and have well-defined sharp thresholds due to more extended destructive interference enabled by the SL periodicity. The electron conductance G and the electron thermal conductance k_e_ follow the energy dependence of the SL transmission coefficient. They show higher peaks than the single QD modulation inside the SL minibands, reaching 1 for completely constructive interference between similar symmetry states of the QDs in the periodic sequence. The Seebeck coefficient shows peaks at the conduction thresholds at the QD minibands and the additional SL minibands. The peaks of S are very much enhanced at the thresholds of the QD minibands. They are even higher than in the case of the two-QD modulation because the thresholds are sharper due to enhanced completely destructive interference between the coherent multiple-QD states of the periodic SL array. The peaks of the Seebeck coefficient at the additional SL minibands are lower. The peaks of the electron transport properties are maximum for the periodic SL. No further enhancement takes place upon adding more identical QDs in the array. The modification of the transmission coefficient and the transport properties occurs for deviations from the periodic SL modulation. We explored the effect of aperiodicity by calculating T_e_, G, k_e_, and S for sequences of six non-identical QDs. The impact of aperiodicity should depend on both the number of the non-identical QDs and their arrangement. Thus, we performed calculations for Fibonacci and random sequences of QDs. We found that aperiodicity distorts the minibands structure, decreasing conductance peaks and shrinking minibands. This is attributed to deviation from the long-range periodicity that limits constructive interference (Figure 6). For the considered degree of disorder, the distortion of the SL minibands is small, and adequate conduction minibands are preserved. In both cases of aperiodic sequence, the peaks of the Seebeck coefficient are significantly lower than in the periodic SL. Notably, additional conduction peaks appear in-between the SL minibands. These are quasi-localized states due to the presence of disorder [6]. They are present for both types of aperiodic sequencies. A comparison between calculations for the Fibonacci and the random sequences shows that the effect of aperiodicity is similar in the two cases. This denotes that the number of non-identical QDs in the modulation profile plays the major role in quantifying the effect of aperiodicity, whereas the arrangement of the QDs plays a secondary role.

**Figure 7:**
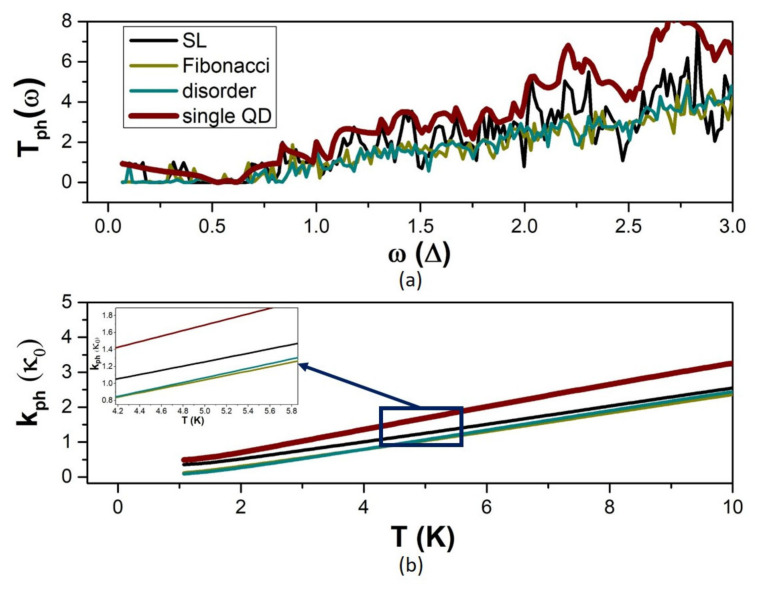
The phonon transport properties in the case of multiple QD-modulation on (**a**) the transmission coefficient versus phonon frequency and (**b**) the phonon thermal conductance versus temperature for: the periodic SL, the aperiodic Fibonacci sequence, and the aperiodic random sequence. The single-QD modulation calculations are also shown for reference.

The phonon transmission coefficient T_ph_ exhibits a richer fluctuation spectrum for the multiple-QD modulation than for the single-QD modulation (Figure 7). In the case of the periodic SL, the fluctuations of T_ph_ follow the fluctuations of the single-QD modulation with sharper deeps. Hence, T_ph_ decreases for the SL sequence, resulting in decrease in the phonon thermal conductance k_ph_. In the case of the aperiodic modulation, T_ph_ shows shorter range fluctuations and decreases in magnitude below the SL values. The thermal conductance k_ph_ of the aperiodic sequences is also smaller than that of the SL. The fluctuations of T_ph_ and the values of k_ph_ are similar in the two cases of aperiodic arrangements. This supports that the effect of disorder on phonon transport is primarily quantified by the number of non-identical QDs in the modulation profile [5]. It can be concluded that the k_ph_ decreases with an increasing number of QD modulation units and that the decrease exceeds the SL decrease for aperiodic sequences of QDs.

**Figure 8:**
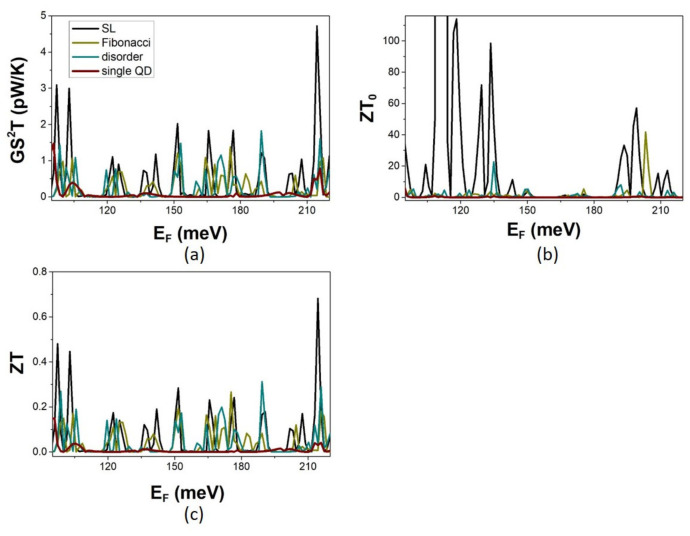
The thermoelectric efficiency in the case of multiple-QD modulation for the SL, the Fibonacci, and the random aperiodic sequences as described in text. (**a**) The energy dependence of the electron transmission coefficient T_e_, and (**b**,**c**) the energy Fermi E_F_, dependence of the conductance G, the electron thermal conductance k_e_, and the Seebeck coefficient S, respectively calculated at T = 5 K for dimensions defined in text. Calculations for the single-QD modulation are shown for reference.

The calculated TE efficiencies for the multiple-QD modulations are shown in Figure 8. The TPF of the multiple-QD sequence is significantly higher than that of the single-QD modulation. It shows a larger enhancement for the periodic SL than for the non-periodic sequences of QDs. It is worth noting that equally high TPF values are shown at the quasi-localized states due to disorder in between the SL minibands. The optimal figure of merit ZT_0_ shows very high peaks at the thresholds of narrow SL minibands where the electron thermal conductance is asymptotically small. The peaks of the ZT are significantly lower due to the dominant phonon contribution to the thermal conductance. ZT is significantly enhanced for multiple-QD modulation compared to the single-QD modulation. The ZT shows the largest enhancement in the case of the SL. Interestingly, the ZT of aperiodic sequences is not deteriorated. At quasi-localized states, ZT shows equally high values as in the SL. Thus, the TE efficiency increases in periodic sequences of modulation units at the edges of minibands or in disordered arrays of QDs at isolated quasi-localized states. Two distinct strategies in enhancing the TE efficiency of MNWs are thereby pointed out.

## 4. Overview and Concluding Remarks

We investigated the effect of increasing modulation degree on the TE efficiency of width-modulated NWs extending gradually the modulation by adding modulation units. Our study was stimulated by the need to understand the effect of multiple modulation units on transport towards optimizing the modulation profile for maximum TE efficiency. First, we studied the case of a two-QD modulation and explored the effect of coupling between two modulation units. Then, we extended our study to the case of multiple-QD modulation and explored the effects of periodic and aperiodic sequences of modulation units. We provide clear new evidence that the TE efficiency depends on the two additional underlying mechanisms, the coupling between the modulation units and the interplay between periodicity and aperiodicity in the modulation profile. Adding more modulation units in the modulation profile increases electron transport properties and the TPF. The increase is maximum for the periodic SL modulation profile. The phonon thermal conductance decreases with increasing modulation, reaching the SL value for periodic modulation. The ZT increases significantly with increasing modulation up to the SL value. Importantly, we showed that a further increase is possible at quasi-localized states in aperiodic modulation profiles where phonon thermal conductance decreases below the SL value and the TPF is comparable to or higher to the SL value. Our results revealed that maximization of the TE power factor is for periodic width modulation, whereas the maximization of the TE efficiency is for aperiodic width-modulation profiles that create quasi-localized states for electrons. Our work indicates new pathways towards optimizing width modulation for maximum TE efficiency when quantum effects dominate transport.

## Data Availability

Data are available from the authors upon reasonable request.
